# Reply: Lymph node density as a surrogate marker for positive lymph nodes

**DOI:** 10.1038/sj.bjc.6606001

**Published:** 2010-11-23

**Authors:** S Polterauer, C Grimm, A Reinthaller, C Tempfer

**Affiliations:** 1 Department of Medicine, Memorial Sloan-Kettering Cancer Center, New York, NY 10065, USA; 2 Department of Gynecology and Gynecologic Oncology, Medical University of Vienna, Vienna, Austria; 3 Department of Gynecology, Medical University of Bochum, Bochum, Germany

**Sir,**

We thank Van Gorp *et al* for their valuable and insightful comments on our study ‘The impact of lymph node density on survival of cervical cancer patients’ (Polterauer *et al*, 2010).

Van Gorp *et al* raise the question whether or not an intra-operative finding of bulky nodes may have biased the lymph node density towards a higher ratio, because the surgeon may have stopped the operation before completing the systematic lymphadenectomy. This is a valuable point. To address this question, we re-analysed our data, looking only at cases with non-bulky nodes. Still, the lymph node density was a prognosticator of disease-free survival (*P*=0.03). This shows that lymph node density, that is, the ratio between positive and removed nodes, is also a valid prognostic marker in women with occult pelvic lymph node metastases.

Second, another valuable criticism of Van Gorp *et al* relates to the influence of the surgeon on the assignment of a given patient to a prognostic group. In our view, this is not ironic, but may rather reflect the biology of the tumour. Obviously, the performance of a surgeon is dependent not only on his/her personal skills, but also on the surgical case itself. Being able to remove a high amount of lymph nodes may reflect the biological behaviour, that is, aggressiveness, of the tumour in the lymph node compartment. Also, women with a constitutively higher number of lymph nodes may be more or less suitable to confront tumour cells migrating to regional lymph nodes on an immunological basis.

Third, the higher median number of lymph nodes removed may truly reflect a learning curve, as suggested by Van Gorp *et al* (Köhler *et al*, 2004). This fact, however, should not be influential on survival, as it affects all cases independently of other prognostic characteristics.

Furthermore, Van Gorp *et al* suggested performing a cross-validation by splitting the data into a training set and a validation set. This computation was not possible, owing to the limited number of patients. Nevertheless, we are currently trying to validate our findings in an independent larger set of data.
